# Current Awareness on Comparative and Functional Genomics

**DOI:** 10.1002/cfg.353

**Published:** 2004-06


**Comparative and Functional Genomics**
*Comp Funct Genom* 2004; **5**: 373–380
Published online in Wiley InterScience (www.interscience.wiley.com). **DOI:** 10.1002/cfg.353

### Current awareness on comparative and functional genomics

CURRENT AWARENESS - USE THE LINKS ONLINE

View this bibliography online at WILEY InterScience®

www.interscience.wiley.com

Your gateway to Wiley journals online

The articles use crossref, the innovative multi-publisher reference linking. This enables readers to move seamlessly from the reference to the cited publication, typically located on a different server and published by a different publisher. Links to MEDLINE abstracts are also available for key journals in the biosciences.

2745

### 1 Reviews & symposia

2003. Special issue: Genomes and evolution. *Curr Opin Genet Dev* **13:** (6)

2003. Special issue: Bead technologies and post-genomic drug discovery. *Comb Chem High Throughput Scr* **6:** (7)

Aebersold R. 2003. A mass spectrometric journey into protein and proteome research. *J Am Soc Mass Spectrom* **14:** (7) 685.

Albertson DG, Pinkel D. 2003. Genomic microarrays in human genetic disease and cancer (Review). *Hum Mol Genet* **12:** (Spec iss 2) R145.

Baltatu O, Bader M. 2003. Brain renin-angiotensin system: Lessons from functional genomics. *Neuroendocrinology* **78:** (5) 253.

Binz PA, Hochstrasser DF, Appel RD. 2003. Mass spectrometry-based proteomics: Current status and potential use in clinical chemistry (Review). *Clin Chem Lab Med* **41:** (12) 1540.

Boonmee S, Imtawil K, Wongkham C, Wongkham S. 2003. Comparative proteomic analysis of juvenile and adult liver fluke, *Opisthorchis viverrini* (Review). *Acta Trop* **88:** (3) 233.

Carlton JM. 2003. Genome sequencing and comparative genomics of tropical disease pathogens (Review). *Cell Microbiol* **5:** (12) 861.

Clarke W, Zhang Z, Chan DW. 2003. The application of clinical proteomics to cancer and other diseases (Review). *Clin Chem Lab Med* **41:** (12) 1562.

Cowman AF, Crabb BS. 2003. Functional genomics: Identifying drug targets for parasitic diseases. *Trends Parasitol* **19:** (11) 538.

Dreger M. 2003. Subcellular proteomics. *Mass Spectrom Rev* **22:** (1) 27.

Gill RT. 2003. Enabling inverse metabolic engineering through genomics. *Curr Opin Biotechnol* **14:** (5) 484.

Grossman AR, Harris EE, Hauser C, Lefebvre PA, Martinez D, Rokhsar D, Shrager J, Silflow CD, Stern D, Vallon O *et al*. 2003. *Chlamydomonas reinhardtii* at the crossroads of genomics (Review). *Eukaryot Cell* **2:** (6) 1137.

Haab BB. 2003. Methods and applications of antibody microarrays in cancer research. *Proteomics* **3:** (11) 2116.

Haberkorn U, Altmann A. 2003. Functional genomics and radioisotope-based imaging procedures. *Ann Med* **35:** (6) 370.

Herrero E, De la Torre MA, Valentin E. 2003. Comparative genomics of yeast species: New insights into their biology. *Int Microbiol* **6:** (3) 183.

Hicks J. 2003. Genome, proteome, and metabolome: Where are we going? *Ultrastruct Pathol* **27:** (5) 289.

Kouprina N, Larionov V*. 2003. Exploiting the yeast *Saccharomyces cerevisiae* for the study of the organization and evolution of complex genomes. *FEMS Microbiol Rev* **27:** (5) 629.

Kumble KD. 2003. Protein microarrays: New tools for pharmaceutical development. *Anal Bioanal Chem* **377:** (5) 812.

Leung YF, Cavalieri D. 2003. Fundamentals of cDNA microarray data analysis (Review). *Trends Genet* **19:** (11) 649.

Lill J. 2003. Proteomic tools for quantitation by mass spectrometry. *Mass Spectrom Rev* **22:** (3) 182.

Lion N, Rohner TC, Dayon L, Arnaud IL, Damoc E, Youhnovski N, Wu ZY, Roussel C, Josserand J, Jensen H *et al*. 2003. Microfluidic systems in proteomics (Review). *Electrophoresis* **24:** (21) 3533.

LoPachin RM, Jones RC, Patterson TA, Slikker W, Barber DS. 2003. Application of proteomics to the study of molecular mechanisms in neurotoxicology (Review). *Neurotoxicology* **24:** (6) 761.

Macarthur DJ, Jacques NA*. 2003. Proteome analysis of oral pathogens (Review). *J Dent Res* **82:** (11) 870.

Marko-Varga G, Nilsson J, Laurell T. 2003. New directions of miniaturization within the proteomics research area (Review). *Electrophoresis* **24:** (21) 3521.

Master SR, Mies C. 2003. Gene-expression profiles and breast-cancer prognosis (Review). *Adv Anat Pathol* **10:** (6) 338.

McGregor E, Dunn MJ*. 2003. Proteomics of heart disease (Review). *Hum Mol Genet* **12:** (Spec iss 2) R135.

Molidor R, Sturn A, Maurer M, Trajanoski Z*. 2003. New trends in bioinformatics: From genome sequence to personalized medicine (Review). *Exp Gerontol* **38:** (10) 1031.

Moritz B, Meyer HE. 2003. Approaches for the quantification of protein concentration ratios. *Proteomics* **3:** (11) 2208.

Peric-Concha N, Long PF. 2003. Mining the microbial metabolome: A new frontier for natural product lead discovery (Review). *Drug Discov Today* **8:** (23) 1078.

Rakwal R, Agrawal GK. 2003. Rice proteomics: Current status and future perspectives. *Electrophoresis* **24:** (19-20) 3378.

Robinson WH, Utz PJ, Steinman L. 2003. Genomic and proteomic analysis of multiple sclerosis: Opinion. *Curr Opin Immunol* **15:** (6) 660.

Sanz-Medel A, Montes-Bayon M, Saanchez MLF. 2003. Trace element speciation by ICP-MS in large biomolecules and its potential for proteomics. *Anal Bioanal Chem* **377:** (2) 236.

Schroder WP, Kieselbach T. 2003. Update on chloroplast proteomics. *Photosynth Res* **78:** (3) 181.

Sebastiani P, Gussoni E, Kohane IS, Ramoni MF. 2003. Statistical challenges in functional genomics. *Stat Sci* **18:** (1) 33.

Baker HV, Churchill GA, Sebastiani P, Gussoni E, Kohane IS, Ramoni MF. 2003. Statistical challenges in functional genomics (Comment on

In order to keep subscribers up-to-date with the latest developments in their field, this current awareness service is provided by John Wiley & Sons and contains newly-published material on comparative and functional genomics. Each bibliography is divided into 16 sections. 1 Reviews & symposia; 2 General; 3 Large-scale sequencing and mapping; 4 Evolutionary genomics; 5 Comparative genomics; 6 Pathways, gene families and regulons; 7 Pharmacogenomics; 8 EST, cDNA and other clone resources; 9 Functional genomics; 10 Transcriptomics; 11 Proteomics; 12 Protein structural genomics; 13 Metabolomics; 14 Genomic approaches to development; 15 Technological advances; 16 Bioinformatics. Within each section, articles are listed in alphabetical order with respect to author. If, in the preceding period, no publications are located relevant to any one of these headings, that section will be omitted.

Copyright © 2004 John Wiley & Sons, Ltd.

above). *Stat Sci* **18:** (1) 60.

Seong SY, Choi CY. 2003. Current status of protein chip development in terms of fabrication and application. *Proteomics* **3:** (11) 2176.

Simon R. 2003. Diagnostic and prognostic prediction using gene expression profiles in high-dimensional microarray data (Review). *Br J Cancer* **89:** (9) 1599.

Sirard MA, Dufort I, Coenen K, Tremblay K, Massicotte L, Robert C. 2002. The use of genomics and proteomics to understand oocyte and early embryo functions in farm animals. *Reproduction* (Suppl 61) 117.

Snell TW, Brogdon SE, Morgan MB. 2003. Gene expression profiling in ecotoxicology. *Ecotoxicology* **12:** (6) 475.

Takahashi N, Yanagida M, Fujiyama S, Hayano T, Isobe T. 2003. Proteomic snapshot analyses of preribosomal ribonucleoprotein complexes formed at various stages of ribosome biogenesis in yeast and mammalian cells. *Mass Spectrom Rev* **22:** (5) 287.

Templin MF, Stoll D, Schwenk JM, Potz O, Kramer S, Joos TO*. 2003. Protein microarrays: Promising tools for proteomic research. *Proteomics* **3:** (11) 2155.

Wang DN. 2003. Carbohydrate microarrays. *Proteomics* **3:** (11) 2167.

Wang HF, Luo XX*. 2003. A-to-I RNA editing: A new mechanism of genomic information modification (Review). *Chin Sci Bull* **48:** (12) 1183.

Witzmann FA, Grant RA. 2003. Pharmacoproteomics in drug development (Review). *Pharmacogenomics J* **3:** (2) 69.

### 3 Large-scale sequencing and mapping

Cerdeno-Tarraga AM, Efstratiou A, Dover LG, Holden MTG, Pallen M, Bentley SD, Besra GS, Churcher C, James KD, De Zoysa A *et al.* 2003. The complete genome sequence and analysis of *Corynebacterium diphtheriae* NCTC13129. *Nucleic Acids Res* **31:** (22) 6516.

Duchaud E, Rusniok C, Frangeul L, Buchrieser C, Givaudan A, Taourit S, Bocs S, Boursaux-Eude C, Chandler M, Charles JF *et al.* 2003. The genome sequence of the entomopathogenic bacterium *Photorhabdus luminescens*. *Nat Biotechnol* **21:** (11) 1307.

Frigaard NU, Chew AGM, Li H, Maresca JA, Bryant DA. 2003. *Chlorobium tepidum*: Insights into the structure, physiology, and metabolism of a green sulfur bacterium derived from the complete genome sequence. *Photosynth Res* **78:** (2) 93.

Gibson-Brown JJ, Osoegawa K, McPherson JD, Waterston RH, De Jong PJ, Rokhsar DS, Holland LZ. 2003. A proposal to sequence the *Amphioxus* genome submitted to the Joint Genome Institute of the US Department of Energy. *J Exp Zool* **300B:** (1) 5.

Healy J, Thomas EE, Schwartz JT, Wigler M. 2003. Annotating large genomes with exact word matches. *Genome Res* **13:** (10) 2306.

Mills R, Rozanov M, Lomsadze A, Tatusova T, Borodovsky M. 2003. Improving gene annotation of complete viral genomes. *Nucleic Acids Res* **31:** (23) 7041.

Nakamura Y, Kaneko T, Sato S, Mimuro M, Miyashita H, Tsuchiya T, Sasamoto S, Watanabe A, Kawashima K, Kishida Y *et al.* 2003. Complete genome structure of *Gloeobacter violaceus* PCC 7421, a cyanobacterium that lacks thylakoids. *DNA Res* **10:** (4) 137.

Palmer LE, Rabinowicz PD, O'Shaughnessy AL, Balija VS, Nascimento LU, Dike S, De la Bastide M, Martienssen RA, McCombie WR. 2003. Maize genome sequencing by methylation filtrations. *Science* **302:** (5653) 2115.

Ren CW, Lee MK, Yan B, Ding KJ, Cox B, Romanov MN, Price JA, Dodgson JB, Zhang HB. 2003. A BAC-based physical map of the chicken genome. *Genome Res* **13:** (12) 2754.

### 4 Evolutionary genomics

Bapteste E, Gribaldo S. 2003. The genome reduction hypothesis and the phylogeny of eukaryotes. *Trends Genet* **19:** (12) 696.

Liu SQ, Guo T, Li XL, Sun ZR. 2003. Bioinformatical study on the proteomics and evolution of SARS-CoV. *Chin Sci Bull* **48:** (13) 1277.

Poustka AJ, Groth D, Hennig S, Thamm S, Cameron A, Beck A, Reinhardt R, Herwig R, Panopoulou G, Lehrach H. 2003. Generation, annotation, evolutionary analysis, and database integration of 20,000 unique sea urchin EST clusters. *Genome Res* **13:** (12) 2736.

Qi Z, Hu Y, Li W, Chen YJ, Zhang ZH, Sun SW, Lu HC, Zhang JF, Bu DB, Ling LJ *et al.* 2003. Phylogeny of SARS-CoV as inferred from complete genome comparison. *Chin Sci Bull* **48:** (12) 1175.

Rokas A, Williams BL, King N, Carroll SB. 2003. Genome-scale approaches to resolving incongruence in molecular phylogenies. *Nature* **425:** (6960) 798.

Volff JN, Bouneau L, Ozouf-Costaz C, Fischer C. 2003. Diversity of retrotransposable elements in compact pufferfish genomes. *Trends Genet* **19:** (12) 674.

Zeigler DR. 2003. Gene sequences useful for predicting relatedness of whole genomes in bacteria. *Int J Syst Evol Microbiol* **53:** (6) 1893.

### 5 Comparative genomics

Chen CY, Wu KM, Chang YC, Chang CH, Tsai HC, Liao TL, Liu YM, Chen HJ, Shen ABT, Li JC *et al.* 2003. Comparative genome analysis of *Vibrio vulnificus*, a marine pathogen. *Genome Res* **13:** (12) 2577.

Pearson BM, Pin C, Wright J, I'Anson K, Humphrey T, Wells JM. 2003. Comparative genome analysis of *Campylobacter jejuni* using whole genome DNA microarrays. *FEBS Lett* **554:** (1-2) 224.

Rio RVM, Lefevre C, Heddi A, Aksoy S. 2003. Comparative genomics of insect-symbiotic bacteria: Influence of host environment on microbial genome composition. *Appl Environ Microbiol* **69:** (11) 6825.

Rodionov DA, Vitreschak AG, Mironov AA, Gelfand MS. 2003. Comparative genomics of the vitamin $B_{12}$ metabolism and regulation in prokaryotes. *J Biol Chem* **278:** (42) 41148.

Stirling B, Yang ZK, Gunter LE, Tuskan GA, Bradshaw HD. 2003. Comparative sequence analysis between orthologous regions of the *Arabidopsis* and *Populus* genomes reveals substantial synteny and microcollinearity. *Can J Forest Res* **33:** (11) 2245.

### 6 Pathways, gene families and regulons

Catlett NL, Yoder OC, Turgeon BG*. 2003. Whole-genome analysis of two-component signal transduction genes in fungal pathogens. *Eukaryot Cell* **2:** (6) 1151.

Erill I, Escribano M, Campoy S, Barbe J. 2003. *In silico* analysis reveals substantial variability in the gene contents of the gamma proteobacteria LexA-regulon. *Bioinformatics* **19:** (17) 2225.

Kho R, Newman JV, Jack RM, Villar HO, Hansen MR. 2003. Genome-wide profile of oxidoreductases in viruses, prokaryotes, and eukaryotes. *J Proteome Res* **2:** (26) 626.

Mashimo T, Glaser P, Lucas M, Simon-Chazottes D, Ceccaldi PE, Montagutelli X, Despres P, Guenet JL. 2003. Structural and functional genomics and evolutionary relationships in the cluster of genes encoding murine 2′,5′-oligoadenylate synthetases. *Genomics* **82:** (5) 537.

### 7 Pharmacogenomics

Amano H, Maruyama K, Naka M, Tanaka T. 2003. Target validation in hypoxia-induced vascular remodeling using transcriptome/metabolome analysis. *Pharmacogenomics J* **3:** (3) 183.

Arai M, Amano S, Ryo A, Hada A, Wakatsuki T, Shuda M, Kondoh N, Yamamoto M. 2003. Identification of epilepsy-related genes by gene expression profiling in the hippocampus of genetically epileptic rat. *Mol Brain Res* **118:** (1-2) 147.

Archacki SR, Angheloiu G, Tian XL, Tan FL, DiPaola N, Shen GQ, Moravec C, Ellis S, Topol EJ, Wang Q. 2003. Identification of new genes differently expressed in coronary artery disease by expression profiling. *Physiol Genomics* **15:** (1) 65.

Arnett HA, Wang Y, Matsushima GK, Suzuki K, Ting JPY. 2003. Functional genomic analysis of remyelination reveals importance of inflammation in oligodendrocyte regeneration. *J Neurosci* **23:** (30) 9824.

Belov L, Huang P, Barber N, Mulligan SP, Christopherson RI. 2003. Identification of repertoires of surface antigens on leukemias using an antibody microarray. *Proteomics* **3:** (11) 2147.

Bernard K, Litman E, Fitzpatrick JL, Shellman YG, Argast G, Polvinen K, Everett AD, Fukasawa K, Norris DA, Alm NG *et al.* 2003. Functional proteomic analysis of melanoma progression. *Cancer Res* **63:** (20) 6716.

Boon K, Edwards JB, Siu IM, Olschner D, Eberhart CG, Marra MA, Strausberg RL, Riggins GJ. 2003. Comparison of medulloblastoma and normal neural transcriptomes identifies a restricted set of activated genes. *Oncogene* **22:** (48) 7687.

Copyright © 2004 John Wiley & Sons, Ltd. 

Bouwman K, Qiu J, Zhou HP, Schotanus M, Mangold LA, Vogt R, Erlandson E, Trenkle J, Partin AW, Misek D *et al*. 2003. Microarrays of tumor cell derived proteins uncover a distinct pattern of prostate cancer serum immunoreactivity. *Proteomics* **3:** (11) 2200.

Brentani H, Caballero OL, Camargo AA, Da Silva AM, Da Silva WA, Neto ED, Grivet M, Gruber A, Guimaraes PEM, Hide W *et al*. 2003. The generation and utilization of a cancer-oriented representation of the human transcriptome by using expressed sequence tags. *Proc Natl Acad Sci U S A* **100:** (23) 13418.

Butterfield DA, Castegna A. 2003. Proteomics for the identification of specifically oxidized proteins in brain: Technology and application to the study of neurodegenerative disorders. *Amino Acids* **25:** (3-4) 419.

Christopher K, Mueller TF, DeFina R, Liang YR, Zhang JH, Gentleman R, Perkins DL. 2003. The graft response to transplantation: A gene expression profile analysis. *Physiol Genomics* **15:** (1) 52.

Decker ED, Zhang YN, Cocklin RR, Witzmann FA, Wang M. 2003. Proteomic analysis of differential protein expression induced by ultraviolet light radiation in HeLa cells. *Proteomics* **3:** (10) 2019.

Deng YX, Xu Y, Ye B, Lei JY, Weinstein MH, O'Leary MP, Richie JP, Mok SC, Liu BCS. 2003. Prostate carcinoma tissue proteomics for biomarker discovery. *Cancer* **98:** (12) 2576.

Dos Remedios CG, Liew CC, Allen PD, Winslow RL, Van Eyk JE, Dunn MJ. 2003. Genomics, proteomics and bioinformatics of human heart failure. *J Muscle Res Cell Motil* **24:** (4-6) 251.

Durier S, Fassot C, Laurent S, Boutouyrie P, Couetil JP, Fine E, Lacolley P, Dzau VJ, Pratt RE. 2003. Physiological genomics of human arteries: Quantitative relationship between gene expression and arterial stiffness. *Circulation* **108:** (15) 1845.

Espina V, Mehta AI, Winters ME, Calvert V, Wulfkuhle J, Petricoin EF, Liotta LA. 2003. Protein microarrays: Molecular profiling technologies for clinical specimens. *Proteomics* **3:** (11) 2091.

Fan QL, Shike T, Shigihara T, Tanimoto M, Gohda T, Makita Y, Wang LN, Horikoshi S, Tomino Y. 2003. Gene expression profile in diabetic KK/Ta mice. *Kidney Int* **64:** (6) 1978.

Fornage M, Swank MW, Boerwinkle E, Doris PA. 2003. Gene expression profiling and functional proteomic analysis reveal perturbed kinase-mediated signaling in genetic stroke susceptibility. *Physiol Genomics* **15:** (1) 75.

Gariboldi M, Spinola M, Milani S, Pignatiello C, Kadota K, Bono H, Hayashizaki Y, Dragani TA, Okazaki Y. 2003. Gene expression profile of normal lungs predicts genetic predisposition to lung cancer in mice. *Carcinogenesis* **24:** (11) 1819.

Ge Y, Molloy MP, Chamberlain JS, Andrews PC. 2003. Proteomic analysis of mdx skeletal muscle: Great reduction of adenylate kinase 1 expression and enzymatic activity. *Proteomics* **3:** (10) 1895.

Grubb RL, Calvert VS, Wulkuhle JD, Paweletz CP, Linehan WM, Phillips JL, Chuaqui R, Valasco A, Gillespie J, Emmert-Buck M *et al*. 2003. Signal pathway profiling of prostate cancer using reverse phase protein arrays. *Proteomics* **3:** (11) 2142.

Grutzmann R, Foerder M, Alldinger I, Staub E, Brummendorf T, Ropcke S, Li XZ, Kristiansen G, Jesnowski R, Sipos B *et al*. 2003. Gene expression profiles of microdissected pancreatic ductal adenocarcinoma. *Virchows Archiv* **443:** (4) 508.

Guo SC, Russo IH, Russo J. 2003. Difference in gene expression profile in breast epithelial cells from women with different reproductive history. *Int J Oncol* **23:** (4) 933.

Hauck SM, Suppmann S, Ueffing M. 2003. Proteomic profiling of primary retinal Muller glia cells reveals a shift in expression patterns upon adaptation to *in vitro* conditions. *Glia* **44:** (3) 251.

Hobbs SK, Shi GY, Homer R, Harsh G, Atlas SW, Bednarski MD. 2003. Magnetic resonance image-guided proteomics of human glioblastoma multiforme. *J Magn Reson Imaging* **18:** (5) 530.

Houtman R, Krijgsveld J, Kool M, Romijn EP, Redegeld FA, Nijkamp FP, Heck AJR, Humphery-Smith I. 2003. Lung proteome alterations in a mouse model for nonallergic asthma. *Proteomics* **3:** (10) 2008.

Hu W, Wu WG, Verschraegen CF, Chen L, Mao L, Yeung SCJ, Kudelka AP, Freedman RS, Kavanagh JJ. 2003. Proteomic identification of heat shock protein 70 as a candidate target for enhancing apoptosis induced by farnesyl transferase inhibitor. *Proteomics* **3:** (10) 1904.

Kaji T, Hachimura S, Kaminogawa S. 2003. Proteome database of unsensitized CD4 positive T-lymphocytes in T-cell receptor transgenic mice. *Electrophoresis* **24:** (19-20) 3433.

Khalyfa A, Klinge CM, Hall WC, Zhao XC, Miller MM, Wang E. 2003. Transcription profiling of estrogen target genes in young and old mouse uterus. *Exp Gerontol* **38:** (10) 1087.

Kim W, Lim SO, Kim JS, Ryu YH, Byeon JY, Kim HJ, Kim YI, Heo JS, Park YM, Jung GH. 2003. Comparison of proteome between hepatitis B virus- and hepatitis C virus-associated hepatocellular carcinoma. *Clin Cancer Res* **9:** (15) 5493.

Latil A, Bieche I, Chene L, Laurendeau I, Berthon P, Cussenot O, Vidaud M. 2003. Gene expression profiling in clinically localized prostate cancer: A four-gene expression model predicts clinical behavior. *Clin Cancer Res* **9:** (15) 5477.

Lee YM, Park SH, Chung KC, Oh YJ. 2003. Proteomic analysis reveals upregulation of calreticulin in murine dopaminergic neuronal cells after treatment with 6-hydroxydopamine. *Neurosci Lett* **352:** (1) 17.

Lehrmann E, Oyler J, Vawter MP, Hyde TM, Kolachana B, Kleinman JE, Huestis ME, Becker KG, Freed WJ. 2003. Transcriptional profiling in the human prefrontal cortex: Evidence for two activational states associated with cocaine abuse. *Pharmacogenomics J* **3:** (1) 27.

Li P, Li J, Feng X, Li Z, Hou R, Han C, Zhang Y. 2003. Gene expression profile of cardiomyocytes in hypertrophic heart induced by continuous norepinephrine infusion in the rats. *Cell Mol Life Sci* **60:** (10) 2200.

Li YW, Ali S, Philip PA, Sarkar FH. 2003. Direct comparison of microarray gene expression profiles between non-amplification and a modified cDNA amplification procedure applicable for needle biopsy tissues. *Cancer Detect Prev* **27:** (5) 405.

Liu XH, Wu YZ, Zehner ZE, Jackson-Cook C, Ware JL. 2003. Proteomic analysis of the tumorigenic human prostate cell line M12 after microcell-mediated transfer of chromosome 19 demonstrates reduction of vimentin. *Electrophoresis* **24:** (19-20) 3445.

Lorenz P, Bantscheff M, Ibrahim SM, Thiesen HJ, Glocker MO. 2003. Proteome analysis of diseased joints from mice suffering from collagen-induced arthritis. *Clin Chem Lab Med* **41:** (12) 1622.

MacKeigan JP, Clements CM, Lich JD, Pope RM, Hod Y, Ting JPY. 2003. Proteomic profiling drug-induced apoptosis in non-small cell lung carcinoma: Identification of RS/DJ-1 and RhoGDI$\alpha$. *Cancer Res* **63:** (20) 6928.

Marcotte ER, Srivastava LK, Quirion R. 2003. cDNA microarray and proteomic approaches in the study of brain diseases: Focus on schizophrenia and Alzheimer's disease. *Pharmacol Ther* **100:** (1) 63.

Miller CJ, Kassem HS, Pepper SD, Hey Y, Ward TH, Margison GP. 2003. Mycoplasma infection significantly alters microarray gene expression profiles. *Biotechniques* **35:** (4) 812.

Moriya T, Seki N, Shimada K, Kato M, Yakushiji T, Nimura Y, Uzawa K, Takiguchi M, Tanzawa H. 2003. In-house cDNA microarray analysis of gene expression profiles involved in SCC cell lines. *Int J Mol Med* **12:** (4) 429.

Nam MJ, Madoz-Gurpide J, Wang H, Lescure P, Schmalbach CE, Zhao R, Misek DE, Kuick DR, Brenner DE, Hanash SM. 2003. Molecular profiling of the immune response in colon cancer using protein microarrays: Occurrence of autoantibodies to ubiquitin C-terminal hydrolase L3. *Proteomics* **3:** (11) 2108.

Nishizuka S, Charboneau L, Young L, Major S, Reinhold WC, Waltham M, Kouros-Mehr H, Bussey KJ, Lee JK, Espina V *et al*. 2003. Proteomic profiling of the NCI-60 cancer cell lines using new high-density reverse-phase lysate microarrays. *Proc Natl Acad Sci U S A* **100:** (24) 14229.

Okada K, Katagiri T, Tsunoda T, Mizutani Y, Suzuki Y, Kamada M, Fujioka T, Shuin T, Miki T, Nakamura Y. 2003. Analysis of gene-expression profiles in testicular seminomas using a genome-wide cDNA microarray. *Int J Oncol* **23:** (6) 1615.

Puchades M, Hansson SF, Nilsson CL, Andreasen N, Blennow K, Davidsson P. 2003. Proteomic studies of potential cerebrospinal fluid protein markers for Alzheimer's disease. *Mol Brain Res* **118:** (1-2) 140.

Rogers MA, Clarke P, Noble J, Munro NP, Paul A, Selby PJ, Banks RE. 2003. Proteomic profiling of urinary proteins in renal cancer by surface enhanced laser desorption ionization and neural-network analysis: Identification of key issues affecting potential clinical utility. *Cancer Res* **63:** (20) 6971.

Sawicki G, Dakour J, Morrish DW. 2003. Functional proteomics of neurokinin B in the placenta indicates a novel role in regulating cytotrophoblast antioxidant defences. *Proteomics* **3:** (10) 2044.

Seth D, Leo MA, McGuinness PH, Lieber CS, Brennan Y, Williams R, Wang XM, McCaughan GW, Gorrell MD, Haber PS. 2003. Gene expression profiling of alcoholic liver disease in the baboon (*Papio hamadryas*) and human liver. *Am J Pathol* **163:** (6) 2303.

Copyright © 2004 John Wiley & Sons, Ltd. 

Shekouh AR, Thompson CC, Prime W, Campbell F, Hamlett J, Herrington CS, Lemoine NR, Crnogorac-Jurcevic T, Buechler MW, Friess H *et al*. 2003. Application of laser capture microdissection combined with two-dimensional electrophoresis for the discovery of differentially regulated proteins in pancreatic ductal adenocarcinoma. *Proteomics* **3:** (10) 1988.

Sidransky D, Irizzary R, Califano JA, Li XB, Ren HN, Benoit N, Mao L. 2003. Serum protein MALDI profiling to distinguish upper aerodigestive tract cancer patients from control subjects. *J Nat Cancer Inst* **95:** (22) 1711.

Somiari RI, Sullivan A, Russell S, Somiari S, Hu H, Jordan R, George A, Katenhusen R, Buchowiecka A, Arciero C *et al*. 2003. High-throughput proteomic analysis of human infiltrating ductal carcinoma of the breast. *Proteomics* **3:** (10) 1863.

Tsoi SM, Cale JM, Bird IM, Kay HH. 2003. cDNA microarray analysis of gene expression profiles in human placenta: Up-regulation of the transcript encoding muscle subunit of glycogen phosphorylase in preeclampsia. *J Soc Gynecol Investig* **10:** (8) 496.

Verrills NM, Walsh BJ, Cobon GS, Hains PG, Kavallaris M. 2003. Proteome analysis of *Vinca* alkaloid response and resistance in acute lymphoblastic leukemia reveals novel cytoskeletal alterations. *J Biol Chem* **278:** (46) 45082.

Wall AM, Rubnitz JE. 2003. Pharmacogenomic effects on therapy for acute lymphoblastic leukemia in children. *Pharmacogenomics J* **3:** (3) 128.

Wong YF, Selvanayagam ZE, Wei N, Porter J, Vittal R, Hu R, Lin Y, Liao J, Shih JW, Cheung TH *et al*. 2003. Expression genomics of cervical cancer: Molecular classification and prediction of radiotherapy response by DNA microarray. *Clin Cancer Res* **9:** (15) 5486.

Wulfkuhle JD, Aquino JA, Calvert VS, Fishman DA, Coukos G, Liotta LA, Petricoin EF. 2003. Signal pathway profiling of ovarian cancer from human tissue specimens using reverse-phase protein microarrays. *Proteomics* **3:** (11) 2085.

Xiao GG, Wang MY, Li N, Loo JA, Nel AE. 2003. Use of proteomics to demonstrate a hierarchical oxidative stress response to diesel exhaust particle chemicals in a macrophage cell line. *J Biol Chem* **278:** (50) 50781.

Yamanaka R, Akutagawa S, Taguchi F, Yajima N, Tsuchiya N, Uzuka T, Morii K, Takahashi H, Tanaka R, Saijo N *et al*. 2003. Selection of surrogate marker genes in primary central nervous system lymphomas for radio-chemotherapy by DNA array analysis of gene expression profiles. *Int J Oncol* **23:** (4) 913.

Young MB, DiSilvestro MR, Sendera TJ, Freund J, Kriete A, Magnuson SR. 2003. Analysis of gene expression in carbon tetrachloride-treated rat livers using a novel bioarray technology. *Pharmacogenomics J* **3:** (1) 41.

Yu HL, Chertkow HM, Bergman H, Schipper HM. 2003. Aberrant profiles of native and oxidized glycoproteins in Alzheimer plasma. *Proteomics* **3:** (11) 2240.

Zhang JP, Ying K, Xiao ZY, Zhou B, Huang QS, Wu HM, Yin M, Xie Y, Mao YM, Rui YC. 2004. Analysis of gene expression profiles in human HL-60 cell exposed to cantharidin using cDNA microarray. *Int J Cancer* **108:** (2) 212.

### 8 EST, cDNA and other clone resources

Clark MS, Edwards YJK, Peterson D, Clifton SW, Thompson AJ, Sasaki M, Suzuki Y, Kikuchi K, Watabe S, Kawakami K *et al*. 2003. Fugu ESTs: New resources for transcription analysis and genome annotation. *Genome Res* **13:** (12) 2747.

Mita K, Morimyo M, Okano K, Koike Y, Nohata J, Kawasaki H, Kadono-Okuda K, Yamamoto Y, Suzuki MG, Shimada T *et al*. 2003. The construction of an EST database for *Bombyx mori* and its application. *Proc Natl Acad Sci U S A* **100:** (24) 14121.

Qiu F, Guo L, Wen TJ, Liu F, Ashlock DA, Schnable PS. 2003. DNA sequence-based “bar codes” for tracking the origins of expressed sequence tags from a maize cDNA library constructed using multiple mRNA sources. *Plant Physiol* **133:** (2) 475.

Roberts SB, Goetz FW. 2003. Expressed sequence tag analysis of gene expressed in the bay scallop, *Argopecten irradians*. *Biol Bull* **205:** (2) 227.

Suh MC, Kim MJ, Hur CG, Bae JM, Park YI, Chung CH, Kang CW, Ohlrogge JB. 2003. Comparative analysis of expressed sequence tags from *Sesamum indicum* and *Arabidopsis thaliana* developing seeds. *Plant Mol Biol* **52:** (6) 1107.

Van Hemert S, Ebbelaar BH, Smits MA, Rebel JMJ. 2003. Generation of EST and microarray resources for functional genomic studies on chicken intestinal health. *Anim Biotechnol* **14:** (2) 133.

Vettore AL, Da Silva FR, Kemper EL, Souza GM, Da Silva AM, Ferro MIT, Henrique-Silva F, Giglioti EA, Lemos MVF, Coutinho LL *et al*. 2003. Analysis and functional annotation of an expressed sequence tag collection for tropical crop sugarcane. *Genome Res* **13:** (12) 2725.

### 9 Functional genomics

Canovas D, Cases I, De Lorenzo V. 2003. Heavy metal tolerance and metal homeostasis in *Pseudomonas putida* as revealed by complete genome analysis. *Environ Microbiol* **5:** (12) 1242.

Cheng KC, Levenson R, Robishaw JD. 2003. Functional genomic dissection of multimeric protein families in zebrafish. *Dev Dyn* **228:** (3) 555.

Dunn W, Chou C, Li H, Hai R, Patterson D, Stolc V, Zhu H, Liu FY. 2003. Functional profiling of a human cytomegalovirus genome. *Proc Natl Acad Sci U S A* **100:** (24) 14223.

Ernest S, Carter M, Hosack A, Rosenblatt D, Ross E, Nadeau JH. 2003. Nutrigenes, functional genomics and systems biology. *J Nutr* **133:** (12) 4267.

Idnurm A, Taylor JL, Pedras MSC, Howlett BJ. 2003. Small scale functional genomics of the blackleg fungus, *Leptosphaeria maculans*: Analysis of a 38 kb region. *Australas Plant Pathol* **32:** (4) 511.

Lee DK, Park JW, Kim YJ, Kim J, Lee Y, Kim J, Kim JS. 2003. Toward a functional annotation of the human genome using artificial transcription factors. *Genome Res* **13:** (12) 2708.

Ohashi Y, Inaoka T, Kasai K, Ito Y, Okamoto S, Satsu H, Tozawa Y, Kawamura F, Ochi K. 2003. Expression profiling of translation-associated genes in sporulating *Bacillus subtilis* and consequence of sporulation by gene inactivation. *Biosci Biotechnol Biochem* **67:** (10) 2245.

Ohlrogge J, Ruuska S, Mekhedov S, Girke T, Benning C. 2003. Functional genomics from a plant biochemist’s perspective. *Acta Agric Scand Sect B Soil Plant* **53:** (Suppl 1) 41.

Potvin E, Lehoux DE, Kukavica-Ibrulj I, Richard KL, Sanschagrin F, Lau GW, Levesque RC. 2003. *In vivo* functional genomics of *Pseudomonas aeruginosa* for high-throughput screening of new virulence factors and antibacterial targets. *Environ Microbiol* **5:** (12) 1294.

Sabina J, Dover N, Templeton LJ, Smulski DR, Soll D, LaRossa RA. 2003. Interfering with different steps of protein synthesis explored by transcriptional profiling of *Escherichia coli* K-12. *J Bacteriol* **185:** (20) 6158.

Siew N, Fischer D. 2003. Analysis of singleton ORFans in fully sequenced microbial genomes. *Protein-Struct Funct Genet* **53:** (2) 241.

Wong SM, Akerley BJ. 2003. Inducible expression system and marker-linked mutagenesis approach for functional genomics of *Haemophilus influenzae*. *Gene* **316:** 177.

### I0 Transcriptomics

Allen TD, Dawe AL, Nuss DL. 2003. Use of cDNA microarrays to monitor transcriptional responses of the chestnut blight fungus *Cryphonectria parasitica* to infection by virulence-attenuating hypoviruses. *Eukaryot Cell* **2:** (6) 1253.

Becher M, Talke IN, Krall L, Kramer U. 2004. Cross-species microarray transcript profiling reveals high constitutive expression of metal homeostasis genes in shoots of the zinc hyperaccumulator *Arabidopsis halleri*. *Plant J* **37:** (2) 251.

Becker JD, Boavida LC, Carneiro J, Haury M, Feijo JA. 2003. Transcriptional profiling of *Arabidopsis* tissues reveals the unique characteristics of the pollen transcriptome. *Plant Physiol* **133:** (2) 713.

Cartieaux F, Thibaud MC, Zimmerli L, Lessard P, Sarrobert C, David P, Gerbaud A, Robaglia C, Somerville S, Nussaume L. 2003. Transcriptome analysis of *Arabidopsis* colonized by a plant-growth promoting rhizobacterium reveals a general effect on disease resistance. *Plant J* **36:** (2) 177.

Dos Reis M, Wernisch L, Savva R. 2003. Unexpected correlations between gene expression and codon usage bias from microarray data for the whole *Escherichia coli* K-12 genome. *Nucleic Acids Res* **31:** (23) 6976.

Copyright © 2004 John Wiley & Sons, Ltd. 

Ekman DR, Lorenz WW, Przybyla AE, Wolfe NL, Dean JFD. 2003. SAGE analysis of transcriptome responses in *Arabidopsis* roots exposed to 2,4,6-trinitrotoluene. *Plant Physiol* **133:** (3) 1397.

Hoth S, Ikeda Y, Morgante M, Wang XJ, Zuo JR, Hanafey MK, Gaasterland T, Tingey SV, Chua NH. 2003. Monitoring genome-wide changes in gene expression in response to endogenous cytokinin reveals targets in *Arabidopsis thaliana*. *FEBS Lett* **554:** (3) 373.

Landi S, Gemignani F, Gioia-Patricola L, Chabrier A, Canzian F. 2003. Evaluation of a microarray for genotyping polymorphisms related to xenobiotic metabolism and DNA repair. *Biotechniques* **35:** (4) 816.

Oh MK, Scoles DR, Haipek C, Strand AD, Gutmann DH, Olson JM, Pulst SM. 2003. Genetic heterogeneity of stably transfected cell lines revealed by expression profiling with oligonucleotide microarrays. *J Cell Biochem* **90:** (5) 1068.

Palma M, Worgall S, Quadri LEN. 2003. Transcriptome analysis of the *Pseudomonas aeruginosa* response to iron. *Arch Microbiol* **180:** (5) 374.

Parsonage G, Falciani F, Burman A, Filer A, Ross E, Bofill M, Martin S, Salmon M, Buckley CD. 2003. Global gene expression profiles in fibroblasts from synovial, skin and lymphoid tissue reveals distinct cytokine and chemokine expression patterns. *Thromb Haemost* **90:** (4) 688.

Pedra JHF, Brandt A, Li HM, Westerman R, Romero-Severson J, Pollack RJ, Murdock LL, Pittendrigh BR. 2003. Transcriptome identification of putative genes involved in protein catabolism and innate immune response in human body louse (Pediculicidae, *Pediculus humanus*). *Insect Biochem Mol Biol* **33:** (11) 1135.

Polissi A, De Laurentis W, Zangrossi S, Briani F, Longhi V, Pesole G, Deho G. 2003. Changes in *Escherichia coli* transcriptome during acclimatization at low temperature. *Res Microbiol* **154:** (8) 573.

Preiss T, Baron-Benhamou J, Ansorge W, Hentze MW. 2003. Homodirectional changes in transcriptome composition and mRNA translation induced by rapamycin and heat shock. *Nat Struct Biol* **10:** (12) 1039.

Sanchez-Carbayo M, Saint F, Lozano JJ, Viale A, Cordon-Cardo C. 2003. Comparison of gene expression profiles in laser-microdissected, nonembedded, and OCT-embedded tumor samples by oligonucleotide microarray analysis. *Clin Chem* **49:** (12) 2096.

Sharma S, Razeghi P, Shakir A, Keneson BJ, Clubb F, Taegtmeyer H. 2003. Regional heterogeneity in gene expression profiles: A transcript analysis in human and rat heart. *Cardiology* **100:** (2) 73.

Van Zhong G, Burns JK. 2003. Profiling ethylene-regulated gene expression in *Arabidopsis thaliana* by microarray analysis. *Plant Mol Biol* **53:** (1) 117.

Zirlinger M. 2003. Selection and validation of microarray candidate genes from subregions and subnuclei of the brain. *Methods* **31:** (4) 290.

### II Proteomics

Ahmed N, Barker G, Oliva K, Garfin D, Talmadge K, Georgiou H, Quinn M, Rice G. 2003. An approach to remove albumin for the proteomic analysis of low abundance biomarkers in human serum. *Proteomics* **3:** (10) 1980.

Andersen JS, Wilkinson CJ, Mayor T, Mortensen P, Nigg EA, Mann M. 2003. Proteomic characterization of the human centrosome by protein correlation profiling. *Nature* **426:** (6966) 570.

Arevalo-Ferro C, Hentzer M, Reil G, Gorg A, Kjelleberg S, Givskov M, Riedel K, Eberl L. 2003. Identification of quorum-sensing regulated proteins in the opportunistic pathogen *Pseudomonas aeruginosa* by proteomics. *Environ Microbiol* **5:** (12) 1350.

Bae MS, Cho EJ, Choi EY, Park OK. 2003. Analysis of the *Arabidopsis* nuclear proteome and its response to cold stress. *Plant J* **36:** (5) 652.

Borderies G, Jamet E, Lafitte C, Rossignol M, Jauneau A, Boudart G, Monsarrat B, Esquerre-Tugaye MT, Boudet A, Pont-Lezica R. 2003. Proteomics of loosely bound cell wall proteins of *Arabidopsis thaliana* cell suspension cultures: A critical analysis. *Electrophoresis* **24:** (19-20) 3421.

Chaurand P, Fouchecourt S, DaGue BB, Xu BGJ, Reyzer ML, Orgebin-Crist MC, Caprioli RM. 2003. Profiling and imaging proteins in the mouse epididymis by imaging mass spectrometry. *Proteomics* **3:** (11) 2221.

Cho YM, Bae SH, Choi BK, Cho SY, Song CW, Yoo JK, Paik YK. 2003. Differential expression of the liver proteome in senescence accelerated mice. *Proteomics* **3:** (10) 1883.

Clack JW, Juhl M, Rice CA, Li JY, Witzmann FA. 2003. Proteomic analysis of transducin β-subunit structural heterogeneity. *Electrophoresis* **24:** (19-20) 3493.

Coleman MA, Miller KA, Beernink PT, Yoshikawa DM, Albala JS. 2003. Identification of chromatin-related protein interactions using protein microarrays. *Proteomics* **3:** (11) 2101.

Cooper B, Eckert D, Andon NL, Yates JR, Haynes PA. 2003. Investigative proteomics: Identification of an unknown plant virus from infected plants using mass spectrometry. *J Am Soc Mass Spectrom* **14:** (7) 736.

Da Cruz S, Xenarios I, Langridge J, Vilbois F, Parone PA, Martinou JC. 2003. Proteomic analysis of the mouse liver mitochondrial inner membrane. *J Biol Chem* **278:** (42) 41566.

Fejes AP, Yi EC, Goodlett DR, Beatty JT. 2003. Shotgun proteomic analysis of a chromatophore-enriched preparation from the purple phototrophic bacterium *Rhodopseudomonas palustris*. *Photosynth Res* **78:** (3) 195.

Franzen B, Yang Y, Sunnemark D, Wickman M, Ottervald J, Oppermann M, Sandberg K. 2003. Dihydropyrimidinase related protein-2 as a biomarker for temperature and time dependent post mortem changes in the mouse brain proteome. *Proteomics* **3:** (10) 1920.

Gallardo K, Le Signor C, Vandekerckhove J, Thompson RD, Burstin J. 2003. Proteomics of *Medicago truncatula* seed development establishes the time frame of diverse metabolic processes related to reserve accumulation. *Plant Physiol* **133:** (2) 664.

Gao J, Opiteck GJ, Friedrichs MS, Dongre AR, Hefta SA. 2003. Changes in the protein expression of yeast as a function of carbon source. *J Proteome Res* **2:** (6) 643.

Gazel A, Ramphal P, Rosdy M, De Wever B, Tornier C, Hosein N, Lee B, Tomic-Canic M, Blumenberg M. 2003. Transcriptional profiling of epidermal keratinocytes: Comparison of genes expressed in skin, cultured keratinocytes, and reconstituted epidermis, using large DNA microarrays. *J Investig Dermatol* **121:** (6) 1459.

Giot L, Bader JS, Brouwer C, Chaudhuri A, Kuang B, Li Y, Hao YL, Ooi CE, Godwin B, Vitols E *et al*. 2003. A protein interaction map of *Drosophila melanogaster*. *Science* **302:** (5651) 1727.

Guina T, Wu MH, Miller SI, Purvine SO, Yi EC, Eng J, Goodlett DR, Aebersold R, Ernst RK, Lee KA. 2003. Proteomic analysis of *Pseudomonas aeruginosa* grown under magnesium limitation. *J Am Soc Mass Spectrom* **14:** (7) 742.

Hakansson K, Emmett MR, Marshall AG, Davidsson P, Nilsson CL. 2003. Structural analysis of 2 D-gel-separated glycoproteins from human cerebrospinal fluid by tandem high-resolution mass spectrometry. *J Proteome Res* **2:** (6) 581.

Heim S, Ferrer M, Heuer H, Regenhardt D, Nimtz M, Timmis KN. 2003. Proteome reference map of *Pseudomonas putida* strain KT2440 for genome expression profiling: Distinct responses of KT2440 and *Pseudomonas aeruginosa* strain PA01 to iron deprivation and a new form of superoxide dismutase. *Environ Microbiol* **5:** (12) 1257.

Helloin E, Jansch L, Phan-Thanh L. 2003. Carbon starvation survival of *Listeria monocytogenes* in planktonic state and in biofilm: A proteomic study. *Proteomics* **3:** (10) 2052.

Ibarrola N, Kalume DE, Gronborg M, Iwahori A, Pandey A. 2003. A proteomic approach for quantitation of phosphorylation using stable isotope labeling in cell culture. *Anal Chem* **75:** (22) 6043.

Jang JH, Hanash S. 2003. Profiling of the cell surface proteome. *Proteomics* **3:** (10) 1947.

Kanski J, Alterman MA, Schoneich C. 2003. Proteomic identification of age-dependent protein nitration in rat skeletal muscle. *Free Radic Biol Med* **35:** (10) 1229.

Kieselbach T, Schroder WP. 2003. The proteome of the chloroplast lumen of higher plants. *Photosynth Res* **78:** (3) 249.

Kohler C, von Eiff C, Peters G, Proctor RA, Hecker M, Engelmann S. 2003. Physiological characterization of a heme-deficient mutant of *Staphylococcus aureus* by a proteomic approach. *J Bacteriol* **185:** (23) 6928.

Li X, Andrews D, Regnier F. 2003. Comparative proteomics of glycoproteins based on lectin selection and isotope coding. *J Proteome Res* **2:** (6) 618.

Malmstrom J, Larsen K, Malmstrom L, Tufvesson E, Parker K, Marchese J, Williamson B, Patterson D, Martin S, Juhasz P, Westergren-Thorsson G, Marko-Varga G. 2003. Nanocapillary liquid chromatography interfaced to tandem matrix-assisted laser desorption/ionization and electrospray ionization-mass spectrometry: Mapping the nuclear

Copyright © 2004 John Wiley & Sons, Ltd. 

proteome of human fibroblasts. *Electrophoresis* **24:** (21) 3806.
Marko-Varga G, Berglund M, Malmstrom J, Lindberg H, Fehniger TE. 2003. Targeting hepatocytes from liver tissue by laser capture microdissection and proteomics expression profiling. *Electrophoresis* **24:** (21) 3800.
Mattow J, Schaible UE, Schmidt F, Hagens K, Siejak R, Brestrich G, Haeselbarth G, Muller EC, Jungblut PR, Kaufmanni SHE. 2003. Comparative proteome analysis of culture supernatant proteins from virulent *Mycobacterium tuberculosis* H37Rv and attenuated *M. bovis* BCG Copenhagen. *Electrophoresis* **24:** (19-20) 3405.
Michaud GA, Salcius M, Zhou F, Bangham R, Bonin J, Guo H, Snyder M, Predki PF, Schweitzer BI. 2003. Analyzing antibody specificity with whole proteome microarrays (Letter). *Nat Biotechnol* **21:** (12) 1509.
Molloy MP, Brzezinski EE, Hang JQ, McDowell MT, Van Bogelen RA. 2003. Overcoming technical variation and biological variation in quantitative proteomics. *Proteomics* **3:** (10) 1912.
Moon MH, Myung S, Plasencia M, Hilderbrand AE, Clemmer DE. 2003. Nanoflow LC/ion mobility/CID/TOF for proteomics: Analysis of a human urinary proteome. *J Proteome Res* **2:** (6) 589.
Orchard S, Zhu WM, Julian RK, Hermjakob H, Apweiler R. 2003. Further advances in the development of a data interchange standard for proteomics data. *Proteomics* **3:** (10) 2065.
Panigrahi AK, Allen TE, Stuart K, Haynes PA, Gygi SP. 2003. Mass spectrometric analysis of the editosome and other multiprotein complexes in *Trypanosoma brucei*. *J Am Soc Mass Spectrom* **14:** (7) 728.
Peyrl A, Krapfenbauer K, Slavc I, Strobel T, Lubec G. 2003. Proteomic characterization of the human cortical neuronal cell line HCN-2. *J Chem Neuroanat* **26:** (3) 171.
Robinson WH, Steinman L, Utz PJ. 2003. Protein arrays for autoantibody profiling and fine-specificity mapping. *Proteomics* **3:** (11) 2077.
Rolland N, Ferro M, Seigneurin-Berny D, Garin J, Douce R, Joyard J. 2003. Proteomics of chloroplast envelope membranes. *Photosynth Res* **78:** (3) 205.
Sazuka T. 2003. Proteomic analysis of the cyanobacterium *Anabaena* sp strain PCC7120 with two-dimensional gel electrophoresis and amino-terminal sequencing. *Photosynth Res* **78:** (3) 279.
Shiio Y, Eisenman RN, Yi EG, Donohoe S, Goodlett DR, Aebersold R. 2003. Quantitative proteomic analysis of chromatin-associated factors. *J Am Soc Mass Spectrom* **14:** (7) 696.
Shimazaki Y, Sugawara Y, Ohtsuka Y, Manabe T. 2003. Analysis of the activity and identification of enzymes after separation of cytosol proteins in mouse liver by microscale nondenaturing two-dimensional electrophoresis. *Proteomics* **3:** (10) 2002.
Shrader EA, Henry TR, Greeley MS, Bradley BP. 2003. Proteomics in zebrafish exposed to endocrine disrupting chemicals. *Ecotoxicology* **12:** (6) 485.
Sickmann A, Reinders J, Wagner Y, Joppich C, Zahedi R, Meyer HE, Schonfisch B, Perschil I, Chacinska A, Guiard B, Rehling P, Pfanner N, Meisinger C. 2003. The proteome of *Saccharomyces cerevisiae* mitochondria. *Proc Natl Acad Sci U S A* **100:** (23) 13207.
Terry DE, Desiderio DM. 2003. Between-gel reproducibility of the human cerebrospinal fluid proteome. *Proteomics* **3:** (10) 1962.
Thome-Kromer B, Bonk I, Klatt M, Nebrich G, Taufmann M, Bryant S, Wacker U, Kopke A. 2003. Toward the identification of liver toxicity markers: A proteome study in human cell culture and rats. *Proteomics* **3:** (10) 1835.
Thongboonkerd V, Klein JB, Arthur JM. 2003. Proteomic identification of a large complement of rat urinary proteins. *Nephron Exp Nephrol* **95:** (2) E69.
Vanrobaeys F, Devreese B, Lecocq E, Rychlewski L, De Smet L, Van Beeumen J. 2003. Proteomics of the dissimilatory iron-reducing bacterium *Shewanella oneidensis* MR-1, using a matrix-assisted laser desorption/ionization-tandem-time of flight mass spectrometer. *Proteomics* **3:** (11) 2249.
Whitelegge JP. 2003. Thylakoid membrane proteomics. *Photosynth Res* **78:** (3) 265.
Woo SH, Kimura M, Higa-Nishiyama A, Dohmae N, Hamamoto H, Jong SK, Yamaguchi I. 2003. Proteome analysis of wheat lemma. *Biosci Biotechnol Biochem* **67:** (11) 2486.
Yao RL, Li JY. 2003. Towards global analysis of mosquito chorion proteins through sequential extraction, two-dimensional electrophoresis and mass spectrometry. *Proteomics* **3:** (10) 2036.
Yoon H, Lim S, Heu S, Choi S, Ryu S. 2003. Proteome analysis of *Salmonella enterica* serovar Typhimurium *fis* mutant. *FEMS Microbiol Lett* **226:** (2) 391.
Zheng SP, Schneider KA, Barder TJ, Lubman DM. 2003. Two-dimensional liquid chromatography protein expression mapping for differential proteomic analysis of normal and O157:H7 *Escherichia coli*. *Biotechniques* **35:** (6) 1202.

### I2 Protein structural genomics

Cammer SA, Hoffman BT, Speir JA, Canady MA, Nelson MR, Knutson S, Gallina M, Baxter SM, Fetrow JS. 2003. Structure-based active site profiles for genome analysis and functional family subclassification. *J Mol Biol* **334:** (3) 387.
Kobe B. 2003. Protein regulation, protein-protein interactions and structural genomics. *Acta Chim Slov* **50:** (3) 547.
Mielke SP, Krishnan VV. 2003. Protein structural class identification directly from NMR spectra using averaged chemical shifts. *Bioinformatics* **19:** (16) 2054.
Shah M, Passovets S, Kim DS, Ellrott K, Wang L, Vokler I, LoCascio P, Xu D, Xu Y. 2003. A computational pipeline for protein structure prediction and analysis at genome scale. *Bioinformatics* **19:** (15) 1985.
Symersky J, Li S, Carson M, Luo D, Luan LH, Luo M. 2003. Structural genomics of *Caenorhabditis elegans*: Structure of dihydropteridine reductase. *Protein-Struct Funct Genet* **53:** (4) 944.
Symersky J, Lin G, Li S, Qiu S, Carson M, Schormann N, Luo M. 2003. Structural genomics of *Caenorhabditis elegans*: Crystal structure of calmodulin. *Protein-Struct Funct Genet* **53:** (4) 947.

### I3 Metabolomics

German JB, Roberts MA, Watkins SM. 2003. Personal metabolomics as a next generation nutritional assessment. *J Nutr* **133:** (12) 4260.
Hang HC, Yu C, Kato DL, Bertozzi CR. 2003. A metabolic labeling approach toward proteomic analysis of mucin-type O-linked glycosylation. *Proc Natl Acad Sci U S A* **100:** (25) 14846.
Hanson TE, Tabita FR. 2003. Insights into the stress response and sulfur metabolism revealed by proteome analysis of a *Chlorobium tepidum* mutant lacking the rubisco-like protein. *Photosynth Res* **78:** (3) 231.

### I4 Genomic approaches to development

Hoyt PR, Doktycz MJ, Beattie KL, Greeley MS. 2003. DNA microarrays detect 4-nonylphenol-induced alterations in gene expression during zebrafish early development. *Ecotoxicology* **12:** (6) 469.
Schmid M, Uhlenhaut NH, Godard F, Demar M, Bressan R, Weigel D, Lohmann JU. 2003. Dissection of floral induction pathways using global expression analysis. *Development* **130:** (24) 6001.
Sparre T, Reusens B, Cherif H, Larsen MR, Roepstorff P, Fey SJ, Larsen PM, Remacle C, Nerup J. 2003. Intrauterine programming of fetal islet gene expression in rats effects of maternal protein restriction during gestation revealed by proteome analysis. *Diabetologia* **46:** (11) 1497.
Tabibiazar R, Wagner RA, Liao A, Quertermous T. 2003. Transcriptional profiling of the heart reveals chamber-specific gene expression patterns. *Circ Res* **93:** (12) 1193.
Thompson LJ, Wang F, Proia AD, Peters KG, Jarrold B, Greis KD. 2003. Proteome analysis of the rat cornea during angiogenesis. *Proteomics* **3:** (11) 2258.
Yu YL, Huang ZY, Yang PY, Rui YC, Yang PY. 2003. Proteomic studies of macrophage-derived foam cell from human U937 cell line using two-dimensional gel electrophoresis and tandem mass spectrometry. *J Cardiovasc Pharmacol* **42:** (6) 782.

### I5 Technological advances

Back P, Nagard F, Bolmsjo G, Bengtsson S, James P. 2003. Automating gel image acquisition. *J Proteome Res* **2:** (6) 662.
Barroso B, Lubda D, Bischoff R. 2003. Applications of monolithic silica capillary columns in proteomics. *J Proteome Res* **2:** (6) 633.
Bodnar WM, Blackburn RK, Krise JM, Moseley MA. 2003. Exploiting the complementary nature of LC/MALDI/MS/MS and LC/ESI/MS/MS

Copyright © 2004 John Wiley & Sons, Ltd. 

for increased proteome coverage. *J Am Soc Mass Spectrom* **14:** (9) 971.
Chamrad DC, Koerting G, Gobom J, Thiele H, Klose J, Meyer HE, Blueggel M. 2003. Interpretation of mass spectrometry data for high-throughput proteomics. *Anal Bioanal Chem* **376:** (7) 1014.
Choi BK, Cho YM, Bae SH, Zoubaulis CC, Paik YK. 2003. Single-step perfusion chromatography with a throughput potential for enhanced peptide detection by matrix-assisted laser desorption/ionization-mass spectrometry. *Proteomics* **3:** (10) 1955.
Coon JJ, Steele HA, Laipis PJ, Harrison WW. 2003. Direct atmospheric pressure coupling of polyacrylamide gel electrophoresis to mass spectrometry for rapid protein sequence analysis. *J Proteome Res* **2:** (6) 610.
Craig R, Krokhin O, Wilkins J, Beavis RC. 2003. Implementation of an algorithm for modeling disulfide bond patterns using mass spectrometry. *J Proteome Res* **2:** (6) 657.
Falk R, Agaton C, Kiesler E, Jin S, Wieslander L, Visa N, Hober S, Stahl S. 2003. An improved dual-expression concept, generating high-quality antibodies for proteomics research. *Biotechnol Appl Biochem* **38:** (3) 231.
Gao XL, Zhou XC, Gulari E. 2003. Light directed massively parallel on-chip synthesis of peptide arrays with t-Boc chemistry. *Proteomics* **3:** (11) 2135.
Guo Z, Xu SY, Lei ZD, Zou HF, Guo BC. 2003. Immobilized metal-ion chelating capillary microreactor for peptide mapping analysis of proteins by matrix assisted laser desorption/ionization-time of flight-mass spectrometry. *Electrophoresis* **24:** (21) 3633.
Hagenstein MC, Mussgnug JH, Lotte K, Plessow R, Brockhinke A, Kruse O, Sewald N. 2003. Affinity-based tagging of protein families with reversible inhibitors: A concept for functional proteomics. *Angew Chem Int Ed* **42:** (45) 5635.
Heller M, Mattou H, Menzel C, Yao XD. 2003. Trypsin catalyzed $^{16}$O-to-$^{18}$O exchange for comparative proteomics: Tandem mass spectrometry comparison using MALDI-TOF, ESI-QTOF, and ESI-ion trap mass spectrometers. *J Am Soc Mass Spectrom* **14:** (7) 704.
Hsu JL, Huang SY, Chow NH, Chen SH. 2003. Stable-isotope dimethyl labeling for quantitative proteomics. *Anal Chem* **75:** (24) 6843.
Kerns RT, Zhang L, Miles MF. 2003. Application of the S-score algorithm for analysis of oligonucleotide microarrays. *Methods* **31:** (4) 274.
Korkola JE, Estep ALH, Pejavar S, DeVries S, Jensen R, Waldman FM. 2003. Optimizing stringency for expression microarrays. *Biotechniques* **35:** (4) 828.
Kuhn K, Thompson A, Prinz T, Muller J, Baumann C, Schmidt G, Neumann T, Hamon C. 2003. Isolation of N-terminal protein sequence tags from cyanogen bromide cleaved proteins as a novel approach to investigate hydrophobic proteins. *J Proteome Res* **2:** (6) 598.
Laugesen S, Roepstorff P. 2003. Combination of two matrices results in improved performance of MALDI MS for peptide mass mapping and protein analysis. *J Am Soc Mass Spectrom* **14:** (9) 992.
Le Bihan T, Duewel HS, Figeys D. 2003. On-line strong cation exchange micro-HPLC-ESI-MS/MS for protein identification and process optimization. *J Am Soc Mass Spectrom* **14:** (7) 719.
Liu DH, Smith DJ. 2003. Voxelation and gene expression tomography for the acquisition of 3-D gene expression maps in the brain. *Methods* **31:** (4) 317.
Lucito R, Healy J, Alexander J, Reiner A, Esposito D, Chi MY, Rodgers L, Brady A, Sebat J, Troge J *et al*. 2003. Representational oligonucleotide microarray analysis: A high-resolution method to detect genome copy number variation. *Genome Res* **13:** (10) 2291.
MacCoss MJ, Wu CC, Liu HB, Sadygov R, Yates JR. 2003. A correlation algorithm for the automated quantitative analysis of shotgun proteomics data. *Anal Chem* **75:** (24) 6912.
Mayfield RD, Liu JW, Randall PK, Lewohl JM, Dodd PR, Harris RA. 2003. Methods for the identification of differentially expressed genes in human post-mortem brain. *Methods* **31:** (4) 301.
Pavlidis P. 2003. Using ANOVA for gene selection from microarray studies of the nervous system. *Methods* **31:** (4) 282.
Phelan ML, Nock S. 2003. Generation of bioreagents for protein chips. *Proteomics* **3:** (11) 2123.
Reese MO, Van Dam RM, Scherer A, Quake SR. 2003. Microfabricated fountain pens for high-density DNA arrays. *Genome Res* **13:** (10) 2348.
Rosengren AT, Salmi JM, Aittokallio T, Westerholm J, Lahesmaa R, Nyman TA, Nevalainen OS. 2003. Comparison of PDQuest and Progenesis software packages in the analysis of two-dimensional electrophoresis gels. *Proteomics* **3:** (10) 1936.
Rubinfeld A, Keren-Lehrer T, Hadas G, Smilansky Z. 2003. Hierarchical analysis of large-scale two-dimensional gel electrophoresis experiments. *Proteomics* **3:** (10) 1930.
Saito S, Matoba R, Kato K. 2003. Adapter-tagged competitive PCR (ATAC-PCR): A high-throughput quantitative PCR method for microarray validation. *Methods* **31:** (4) 326.
Schweitzer B, Predki P, Snyder M. 2003. Microarrays to characterize protein interactions on a whole-proteome scale. *Proteomics* **3:** (11) 2190.
Smyth GK, Speed T. 2003. Normalization of cDNA microarray data. *Methods* **31:** (4) 265.
Strittmatter EF, Ferguson PL, Tang KQ, Smith RD. 2003. Proteome analyses using accurate mass and elution time peptide tags with capillary LC time-of-flight mass spectrometry. *J Am Soc Mass Spectrom* **14:** (9) 980.
Suckau D, Resemann A, Schuerenberg M, Hufnagel P, Franzen J, Holle A. 2003. A novel MALDI LIFT-TOF/TOF mass spectrometer for proteomics. *Anal Bioanal Chem* **376:** (7) 952.
Ting AC, Lee SF, Wang K. 2003. An easy setup for double slide microarray hybridization. *Biotechniques* **35:** (4) 808.
Wurmbach E, Yuen T, Sealfon SC. 2003. Focused microarray analysis. *Methods* **31:** (4) 306.
Zhang DM, Xie Y, Mrozek MF, Ortiz C, Davisson VJ, Ben-Amotz D. 2003. Raman detection of proteomic analytes. *Anal Chem* **75:** (21) 5703.
Zhang S, Van Pelt CK, Henion JD. 2003. Automated chip-based nanoelectrospray-mass spectrometry for rapid identification of proteins separated by two-dimensional gel electrophoresis. *Electrophoresis* **24:** (21) 3620.
Zhang WZ, Krutchinsky AN, Chait BT. 2003. "*De novo*" peptide sequencing by MALDI-quadrupole-ion trap mass spectrometry: A preliminary study. *J Am Soc Mass Spectrom* **14:** (9) 1012.

### I6 Bioinformatics

Abril JF, Guigo R, Wiehe T. 2003. gff2aplot: Plotting sequence comparisons. *Bioinformatics* **19:** (18) 2477.
Ahola V, Aittokallio T, Uusipaikka E, Vihinen M. 2003. Efficient estimation of emission probabilities in profile hidden Markov models. *Bioinformatics* **19:** (18) 2359.
Argraves GL, Barth JL, Argraves WS. 2003. The MUSC DNA microarray database. *Bioinformatics* **19:** (18) 2473.
Bader JS. 2003. Greedily building protein networks with confidence. *Bioinformatics* **19:** (15) 1869.
Berriz GF, King OD, Bryant B, Sander C, Roth FP. 2003. Characterizing gene sets with FuncAssociate. *Bioinformatics* **19:** (18) 2502.
Bhagwat AA, Phadke RP, Wheeler D, Kalantre S, Gudipati M, Bhagwat M. 2003. Computational methods and evaluation of RNA stabilization reagents for genome-wide expression studies. *J Microbiol Methods* **55:** (2) 399.
Bolshakova N, Azuaje F. 2003. Machaon CVE: Cluster validation for gene expression data. *Bioinformatics* **19:** (18) 2494.
Boulesteix AL, Tutz G, Strimmer K. 2003. A CART-based approach to discover emerging patterns in microarray data. *Bioinformatics* **19:** (18) 2465.
Brugger K, Redder P, Skovgaard M. 2003. MUTAGEN: Multi-User Tool for Annotating GENomes. *Bioinformatics* **19:** (18) 2480.
Carbone A, Zinovyev A, Kepes F. 2003. Codon adaptation index as a measure of dominating codon bias. *Bioinformatics* **19:** (16) 2005.
Carter K, Bellgard M. 2003. MASV-Multiple (BLAST) Annotation System Viewer. *Bioinformatics* **19:** (17) 2313.
Chen ZR. 2003. Assessing sequence comparison methods with the average precision criterion. *Bioinformatics* **19:** (18) 2456.
Choi V, Farach-Colton M. 2003. Barnacle: An assembly algorithm for clone-based sequences of whole genomes. *Gene* **320:** 165.
Daub CO, Kloska S, Selbig J. 2003. MetaGeneAlyse: Analysis of integrated transcriptional and metabolite data. *Bioinformatics* **19:** (17) 2332.
De Boer WPH, Oudejans JJ, Meijer CJLM, Lankelma J. 2003. Analysing gene expressions with GRANK. *Bioinformatics* **19:** (15) 2000.
De la Nava JG, Van Hijum S, Trelles O. 2003. *Pre*P: Gene expression data pre-processing. *Bioinformatics* **19:** (17) 2328.
Eichler GS, Huang S, Ingber DE. 2003. Gene Expression Dynamics In-

Copyright © 2004 John Wiley & Sons, Ltd. 

spector (GEDI): For integrative analysis of expression profiles. *Bioinformatics* **19:** (17) 2321.
Fang Z, Cone K, Sanchez-Villeda H, Polacco M, McMullen M, Schroeder S, Gardiner J, Davis G, Havermann S, Yim Y *et al*. 2003. iMap: A database-driven utility to integrate and access the genetic and physical maps of maize. *Bioinformatics* **19:** (16) 2105.
Gat-Viks I, Sharan R, Shamir R. 2003. Scoring clustering solutions by their biological relevance. *Bioinformatics* **19:** (18) 2381.
Giles PJ, Kipling D. 2003. Normality of oligonucleotide microarray data and implications for parametric statistical analyses. *Bioinformatics* **19:** (17) 2254.
Gille C, Lorenzen S, Michalsky E, Frommel C. 2003. KISS for STRAP: User extensions for a protein alignment editor. *Bioinformatics* **19:** (18) 2489.
Gomez SM, Noble WS, Rzhetsky A. 2003. Learning to predict protein-protein interactions from protein sequences. *Bioinformatics* **19:** (15) 1875.
Gordon L, Chervonenkis AY, Gammerman AJ, Shahmuradov IA, Solovyev VV. 2003. Sequence alignment kernel for recognition of promoter regions. *Bioinformatics* **19:** (15) 1964.
Han K, Ju BH. 2003. A fast layout algorithm for protein interaction networks. *Bioinformatics* **19:** (15) 1882.
Hautaniemi S, Edgren H, Vesanen P, Wolf M, Jarvinen AK, Yli-Harja O, Astola J, Kallioniemi O, Monni O. 2003. A novel strategy for microarray quality control using Bayesian networks. *Bioinformatics* **19:** (16) 2031.
Hill A, Kim H. 2003. The UAB Proteomics Database. *Bioinformatics* **19:** (16) 2149.
Hsu AL, Tang SL, Halgamuge SK. 2003. An unsupervised hierarchical dynamic self-organizing approach to cancer class discovery and marker gene identification in microarray data. *Bioinformatics* **19:** (16) 2131.
Huang XH, Pan W. 2003. Linear regression and two-class classification with gene expression data. *Bioinformatics* **19:** (16) 2072.
Hummon AB, Hummon NP, Corbin RW, Li LJ, Vilim FS, Weiss KR, Sweedler JV. 2003. From precursor to final peptides: A statistical sequence-based approach to predicting prohormone processing. *J Proteome Res* **2:** (6) 650.
Husmeier D. 2003. Sensitivity and specificity of inferring genetic regulatory interactions from microarray experiments with dynamic Bayesian networks. *Bioinformatics* **19:** (17) 2271.
Iacovoni JS. 2003. GeneHuggers: Database mining and application connectivity tools for subsequence analyses of the human genome. *Bioinformatics* **19:** (17) 2316.
Jain N, Thatte J, Braciale T, Ley K, O'Connell M, Lee JK. 2003. Local-pooled-error test for identifying differentially expressed genes with a small number of replicated microarrays. *Bioinformatics* **19:** (15) 1945.
Kankainen M, Wong G. 2003. DANCER: A program for digital anatomical reconstruction of gene expression data - Online art. no. e132. *Nucleic Acids Res* **31:** (21) e132.
Karev GP, Wolf YI, Koonin EV. 2003. Simple stochastic birth and death models of genome evolution: Was there enough time for us to evolve? *Bioinformatics* **19:** (15) 1889.
Khan S, Situ G, Decker K, Schmidt CJ. 2003. GoFigure: Automated Gene Ontology™ annotation. *Bioinformatics* **19:** (18) 2484.
Krause R, Von Mering C, Bork P. 2003. A comprehensive set of protein complexes in yeast: Mining large scale protein-protein interaction screens. *Bioinformatics* **19:** (15) 1901.
Krishnan VG, Westhead DR. 2003. A comparative study of machine-learning methods to predict the effects of single nucleotide polymorphisms on protein function. *Bioinformatics* **19:** (17) 2199.
Landais I, Ogliastro M, Mita K, Nohata J, Lopez-Ferber M, Duonor-Cerutti M, Shimada T, Fournier P, Devauchelle G. 2003. Annotation pattern of ESTs from *Spodoptera frugiperda* Sf9 cells and analysis of the ribosomal protein genes reveal insect-specific features and unexpectedly low codon usage bias. *Bioinformatics* **19:** (18) 2343.
Lee DY, Yun H, Park S, Lee SY. 2003. MetaFluxNet: The management of metabolic reaction information and quantitative metabolic flux analysis. *Bioinformatics* **19:** (16) 2144.
Lexa M, Valle G. 2003. PRIMEX: Rapid identification of oligonucleotide matches in whole genomes. *Bioinformatics* **19:** (18) 2486.
Liu WM, Di XJ, Yang G, Matsuzaki H, Huang J, Mei R, Ryder TB, Webster TA, Dong SL, Liu GY *et al*. 2003. Algorithms for large-scale genotyping microarrays. *Bioinformatics* **19:** (18) 2397.
Liu XL, Muller HG. 2003. Modes and clustering for time-warped gene expression profile data. *Bioinformatics* **19:** (15) 1937.
Lohmann KK, Von der Lieth CW. 2003. GLYCO-FRAGMENT: A web tool to support the interpretation of mass spectra of complex carbohydrates. *Proteomics* **3:** (10) 2028.
Lukashin AV, Lukashev ME, Fuchs R. 2003. Topology of gene expression networks as revealed by data mining and modeling. *Bioinformatics* **19:** (15) 1909.
Martinez-Cruz LA, Rubio A, Martinez-Chantar ML, Labarga A, Barrio I, Podhorski A, Segura V, Campo JLS, Avila MA, Mato JM. 2003. GARBAN: Genomic analysis and rapid biological annotation of cDNA microarray and proteomic data. *Bioinformatics* **19:** (16) 2158.
Michailidis G, Shedden K. 2003. The application of rule-based methods to class prediction problems in genomics. *J Comput Biol* **10:** (5) 689.
Narasimhan C, LoCascio P, Uberbacher E. 2003. Background rareness-based iterative multiple sequence alignment algorithm for regulatory element detection. *Bioinformatics* **19:** (15) 1952.
Nikitin A, Egorov S, Daraselia N, Mazo I. 2003. Pathway studio: The analysis and navigation of molecular networks. *Bioinformatics* **19:** (16) 2155.
Oba S, Sato M, Takemasa I, Monden M, Matsubara K, Ishii S. 2003. A Bayesian missing value estimation method for gene expression profile data. *Bioinformatics* **19:** (16) 2088.
Otu HH, Sayood K. 2003. A new sequence distance measure for phylogenetic tree construction. *Bioinformatics* **19:** (16) 2122.
Pegg SCH, Novak WRP, Babbitt PC. 2003. Intersect: Identification and visualization of overlaps in database search results. *Bioinformatics* **19:** (15) 1997.
Qian J, Lin J, Luscombe NM, Yu HY, Gerstein M. 2003. Prediction of regulatory networks: Genome-wide identification of transcription factor targets from gene expression data. *Bioinformatics* **19:** (15) 1917.
Qin J, Lewis DP, Noble WS. 2003. Kernel hierarchical gene clustering from microarray expression data. *Bioinformatics* **19:** (16) 2097.
Rocco D, Critchlow T. 2003. Automatic discovery and classification of bioinformatics Web sources. *Bioinformatics* **19:** (15) 1927.
Rojdestvenski I. 2003. Metabolic pathways in three dimensions. *Bioinformatics* **19:** (18) 2436.
Roset R, Subirana JA, Messeguer X. 2003. MREPATT: Detection and analysis of exact consecutive repeats in genomic sequences. *Bioinformatics* **19:** (18) 2475.
Sanchez-Villeda H, Schroeder S, Polacco M, McMullen M, Havermann S, Davis G, Vroh-Bi I, Cone K, Sharopova N, Yim Y *et al*. 2003. Development of an integrated laboratory information management system for the maize mapping project. *Bioinformatics* **19:** (16) 2022.
Smid M, Dorssers LCJ, Jenster G. 2003. Venn Mapping: Clustering of heterologous microarray data based on the number of co-occurring differentially expressed genes. *Bioinformatics* **19:** (16) 2065.
Thareau V, Dehais P, Serizet C, Hilson P, Rouze P, Aubourg S. 2003. Automatic design of gene-specific sequence tags for genome-wide functional studies. *Bioinformatics* **19:** (17) 2191.
Thornton K. 2003. libsequence: A C++ class library for evolutionary genetic analysis. *Bioinformatics* **19:** (17) 2325.
Vencio RZN, Brentani H, Pereira CAB. 2003. Using credibility intervals instead of hypothesis tests in SAGE analysis. *Bioinformatics* **19:** (18) 2461.
Vinogradov AE. 2003. Silent DNA: Speaking RNA language? *Bioinformatics* **19:** (17) 2167.
Vohradsky J, Janda I, Grunenfelder B, Berndt P, Roder D, Langen H, Weiser J, Jenal U. 2003. Proteome of *Caulobacter crescentus* cell cycle publicly accessible on SWICZ server. *Proteomics* **3:** (10) 1874.
Wang H, Ooi BC, Tan KL, Ong TH, Zhou L. 2003. BLAST++: BLASTing queries in batches. *Bioinformatics* **19:** (17) 2323.
Wang JB, Myklebost O, Hovig E. 2003. MGraph: Graphical models for microarray data analysis. *Bioinformatics* **19:** (17) 2210.
Wang YH, Liu JW, Zhao TJ, Ji Q. 2003. Recognizing translation initiation sites of eukaryotic genes based on the cooperatively scanning model. *Bioinformatics* **19:** (15) 1972.
Wu W, Roberts SLL, Armitage JR, Tooke P, Cordingley HC, Wildsmith SE. 2003. Validation of consensus between proteomic and clinical chemistry datasets by applying a new randomisation F-test for generalised procrustes analysis. *Anal Chim Acta* **490:** (1-2) 365.
Zhou XB, Wang XD, Dougherty ER. 2003. Missing-value estimation using linear and non-linear regression with Bayesian gene selection. *Bioinformatics* **19:** (17) 2302.

Copyright © 2004 John Wiley & Sons, Ltd. 
